# Structural and Thermal Assessment of POE Encapsulant Residues from Laser-Treated Photovoltaic Laminate Fragments

**DOI:** 10.3390/polym18141785

**Published:** 2026-07-21

**Authors:** Szymon Tofil, Shuyang Lin, Jianhua Yao, Qunli Zhang, Liang Wang, Leonardo Orazi, António B. Pereira, Filipe J. Oliveira

**Affiliations:** 1 Institute of Laser Advanced Manufacturing, Zhejiang University of Technology, No. 288 Liuhe Road, Liuxia Subdistrict, Xihu District, Hangzhou 310023, China; 2 Moganshan Institute, Zhejiang University of Technology, Kangqian District, Deqing 313200, China; 3 Laser Processing Research Center, Faculty of Mechatronics and Mechanical Engineering, Kielce University of Technology, 7, Av. Tysiąclecia P.P., 25-314 Kielce, Poland; 4 Department of Sciences and Methods for Engineering, Università degli Studi di Modena e Reggio Emilia, 42122 Reggio Emilia, Italy; 5 TEMA—Centre for Mechanical Technology and Automation, Department of Mechanical Engineering, University of Aveiro, Campus de Santiago, 3810-193 Aveiro, Portugal; 6 CICECO—Aveiro Institute of Materials, Department of Materials and Ceramic Engineering, University of Aveiro, Campus Universitário de Santiago, 3810-193 Aveiro, Portugal

**Keywords:** photovoltaic waste, POE encapsulant, polyolefin elastomer, FTIR-ATR, TGA/DTG, SEM/EDS, inorganic residues, silicon photovoltaic recycling, polymer-rich residues

## Abstract

End-of-life photovoltaic modules represent a complex waste stream in which polyolefin elastomer (POE) encapsulants are increasingly important but insufficiently characterized after recovery. This study evaluates the structural, morphological and thermal state of POE-rich encapsulant residues obtained from laser-treated fragments of a crystalline-silicon photovoltaic module after approximately five years of field operation, focusing on material quality rather than process optimization. Reference POE and representative polymer-rich residues were examined by FTIR-ATR, TGA/DTG under nitrogen and SEM/EDS. FTIR-ATR showed characteristic polyolefin bands at approximately 2915–2847, 1463 and 718–719 cm^−1^ in both reference POE and treated residues, indicating retention of the hydrocarbon backbone. Treated residues exhibited additional features in the 1800–1500 and 1100–1000 cm^−1^ regions, attributed to oxygen-containing surface species and interfacial glass/silicon contributions. TGA/DTG revealed a similar main decomposition range for the POE-rich residues, with DTG peaks mainly between 471.5 and 474.3 °C, while residual mass varied from 1.94 to 23.03%, compared with 0.04% for reference POE. SEM/EDS confirmed heterogeneous surfaces and local silicon/oxygen-rich particles attached to or embedded in the polymer-rich residues. The results show that POE-rich waste fractions can preserve the main polyolefin structure, but their valorization requires control of inorganic contamination, especially for cut, cracked or mechanically damaged modules.

## 1. Introduction

The rapid expansion of photovoltaic energy has created an increasing need for efficient end-of-life management of solar modules. Although other sustainable energy-conversion technologies, including thermoelectric and moisture–electric systems, are also being actively developed, photovoltaic technology is already deployed at a scale that creates a near-term and rapidly growing end-of-life materials management challenge [1,2]. Crystalline silicon photovoltaic modules are multi-material structures composed mainly of glass, silicon cells, metallic conductors, encapsulants and polymeric backsheets. Their layered architecture ensures long-term operational stability, but at the same time makes selective recovery of individual material fractions difficult [3,4,5,6,7,8].

Polymeric encapsulants are among the most critical components of photovoltaic modules. They provide mechanical stabilization of the cells, electrical insulation, optical coupling and protection against moisture and environmental stress. For decades, ethylene-vinyl acetate was the dominant encapsulation material; however, modern module designs increasingly use polyolefin elastomers and thermoplastic polyolefin-based encapsulants. This transition is driven by the improved hydrolytic stability of POE, reduced water uptake and elimination of acetic acid-related corrosion pathways, which are particularly important for high-efficiency cell technologies such as heterojunction and TOPCon architectures [9,10,11,12].

Despite these advantages, POE encapsulants introduce new challenges for recycling. The encapsulant is typically crosslinked or strongly bonded to glass and silicon cell interfaces, often through silane-based adhesion promoters or interfacial coupling mechanisms. As a result, recovered polymer-rich fractions from real photovoltaic modules are rarely chemically pure materials. They may contain glass fragments, silicon cell particles, metallic residues, interfacial layers and products of long-term environmental ageing [12,13,14,15].

A growing number of studies have investigated mechanical, thermal, chemical, water-jet-assisted and laser-assisted routes for photovoltaic module disassembly and material recovery [16,17,18,19,20,21,22,23]. These works demonstrate that physical separation routes can enable selective access to valuable module components. However, less attention has been paid to the material state of the recovered encapsulant-rich residue itself, especially for POE-based encapsulants. From the perspective of further valorization, it is essential to determine whether the recovered fraction retains the main polyolefin structure, whether its thermal stability is affected, and how strongly the interpretation of analytical data is influenced by residual glass and silicon fragments.

FTIR-ATR spectroscopy is widely used for identifying functional groups and degradation markers in photovoltaic encapsulants, including carbonyl-containing species, hydroxyl groups and changes in aliphatic C-H bands [9,14,24,25]. TGA/DTG provides complementary information on thermal stability, decomposition temperature ranges and residual inorganic content [26,27,28]. SEM/EDS can further support the interpretation by visualizing surface morphology and confirming the elemental composition of inorganic residues attached to polymer-rich fractions.

The objective of this work is to evaluate the chemical structure, morphology and thermal behaviour of POE-rich encapsulant residues obtained from laser-treated photovoltaic laminate fragments. In contrast to studies focused on process parameters for layer separation, the present work concentrates on the material characteristics of the recovered polymer-rich fraction. The analysis combines FTIR-ATR, TGA/DTG and SEM/EDS to distinguish between changes in the POE matrix and the contribution of inorganic residues originating from glass and silicon cell fragments.

## 2. Materials and Methods

### 2.1. Materials and Sample Selection

The investigated material consisted of POE-rich encapsulant residues collected from a commercial crystalline silicon photovoltaic module that had operated under field conditions for approximately five years before sample preparation. The module was manufactured by Jinzhou Yangguang Jinmao Photovoltaic Technology Co., Ltd. (Solariga Energy, Jinzhou, China) and was identified as a crystalline silicon photovoltaic module, model JMPV-6DM/72-360, with a nominal maximum power of 360 W under standard test conditions. The module label indicated compliance with IEC 61215 and IEC 61730 standards. Complete service-history data and detailed degradation records were not available; therefore, the material was treated as a representative field-aged commercial PV laminate rather than as a module with a fully documented degradation history. The residues were obtained from laminate fragments after controlled laser treatment. The samples represented realistic polymer-rich waste fractions rather than chemically purified POE. Therefore, local contamination by glass fragments, silicon cell residues and interfacial inorganic particles was expected and was considered an inherent feature of the investigated material stream. The selection of laser-treated samples was motivated by the growing interest in photonics-based, solvent-free and non-contact routes for photovoltaic module disassembly, which require a dedicated assessment of the resulting material state.

**Figure 1:**
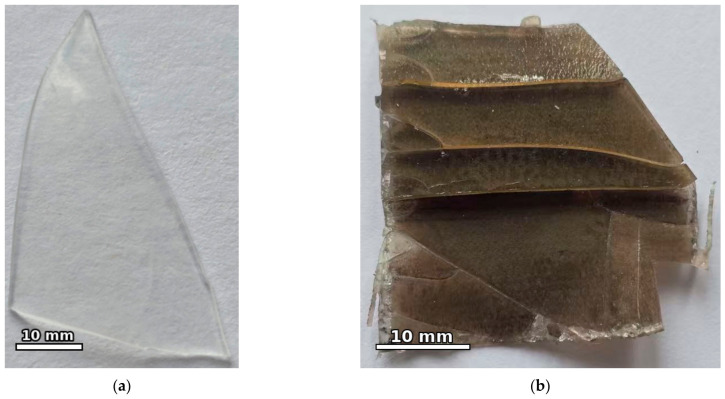

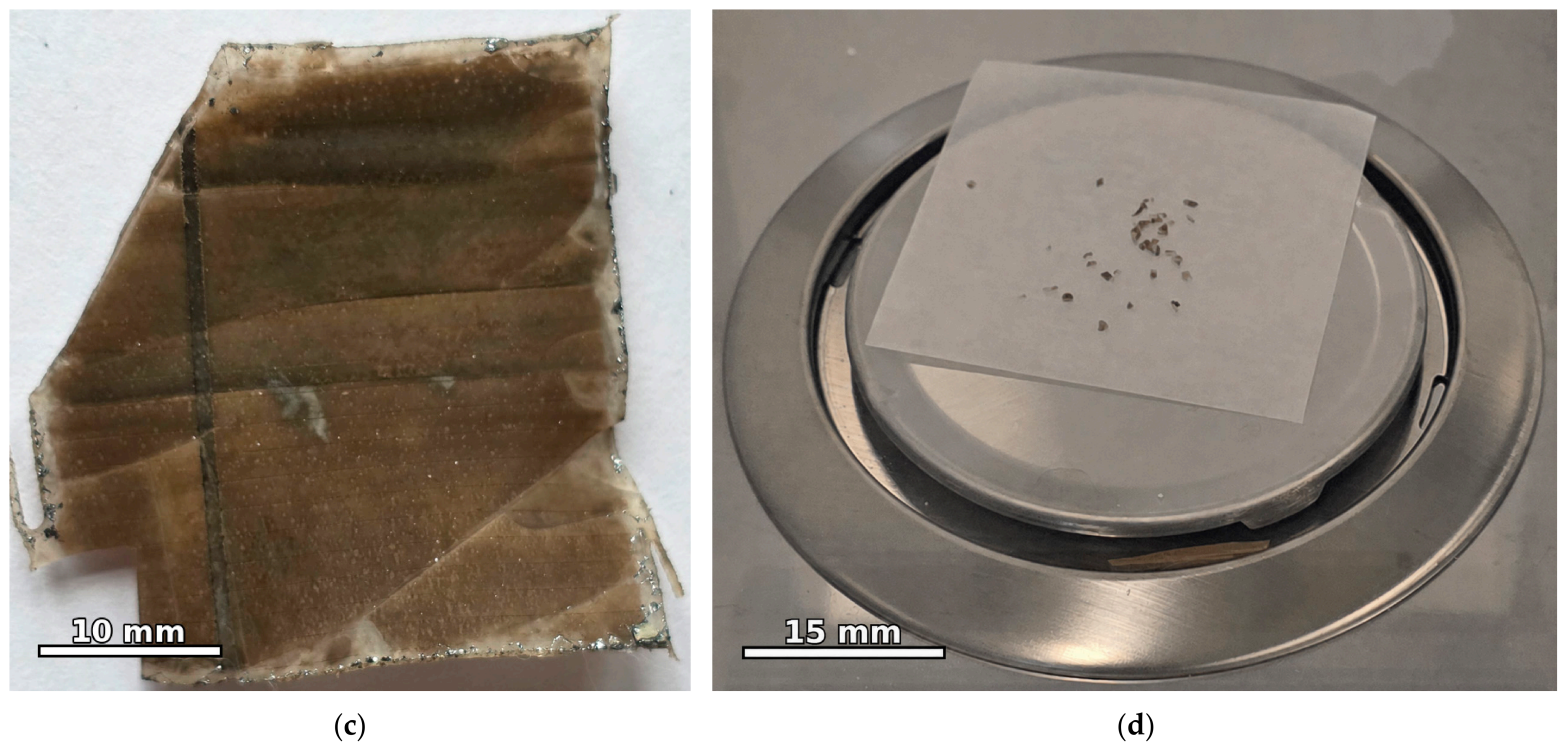
Representative POE-rich residues collected from photovoltaic laminate fragments: (**a**) reference POE film; (**b**) POE-rich residue with visually distinct dark and white regions and visible glass particles; (**c**) POE-rich residue containing visible silicon cell fragments; (**d**) representative sample fragments after preparation for TGA/DTG analysis. The photographed laminate fragment had approximate in-plane dimensions of 33.5 × 34.2 mm, as documented using a digital calliper. For TGA/DTG, smaller fragments were collected from representative regions and analyzed by mass-normalized thermogravimetry. The images illustrate the heterogeneous nature of the investigated material and confirm that the samples represent realistic POE-rich waste fractions rather than chemically purified polymer.

The analyzed samples included surfaces associated with the glass-facing side and the silicon cell-facing side of the encapsulant, as well as visually distinct dark and white regions. Representative examples are shown in Figure 1. Additional samples obtained under lower exposure conditions and different scanning speeds were included to provide comparative material states. A reference POE encapsulant film, not subjected to laser treatment, was used as the baseline material. The encapsulant thickness was not treated as an independent variable in this work. In commercial laminated PV modules, encapsulant layers are formed under controlled pressure and temperature during module manufacturing, leading to nominally uniform layer thickness within the laminate. Moreover, the present study did not evaluate optical transmission or bulk optical absorption. FTIR-ATR was used as a surface-sensitive technique, TGA/DTG data were normalized to initial sample mass, and SEM/EDS was used to assess local surface morphology and elemental composition.

To clarify the sample nomenclature, the codes used in this work refer to selected material states obtained under different nominal laser-exposure conditions and to the local region from which the residue was collected. The sample codes were used only for traceability of the analytical specimens and should not be interpreted as a process-optimization matrix. The detailed laser-processing strategy is outside the scope of the present work; however, the corresponding nominal cumulative fluence values and analytical use of each sample group are summarized in Table 1.

The nominal cumulative fluence was calculated as the surface energy dose delivered during raster scanning, using the average laser power, scanning speed, number of passes and line spacing. This value was used only as a compact descriptor of the laser-exposure level and does not represent a complete process recipe or optimization parameter set.

### 2.2. FTIR-ATR Spectroscopy

FTIR-ATR spectra were recorded using a Nicolet Apex FT-IR spectrometer (Thermo Fisher Scientific, Waltham, MA, USA) equipped with an ID7/ITX ATR accessory with a diamond crystal. The spectra were collected in the range of 4000–400 cm^−1^ with a spectral resolution of 4 cm^−1^ and 32 scans per spectrum.

The analysis focused on the main spectral regions associated with polyolefin structure and surface/interfacial modifications: 3000–2800 cm^−1^ for aliphatic C-H stretching vibrations, 1800–1500 cm^−1^ for carbonyl-related and unsaturated/interfacial bands, 1500–1300 cm^−1^ for CH_2_/CH_3_ deformation vibrations, 1100–1000 cm^−1^ for C-O/Si-O-related contributions, and approximately 718–720 cm^−1^ for CH_2_ rocking vibrations.

Because the reference POE already exhibited a carbonyl-related band near 1733 cm^−1^, interpretation of the treated residues was based not on the simple presence of this band, but on changes in band shape, relative intensity and the contribution of additional spectral components.

To provide a semi-quantitative comparison of carbonyl/interfacial contributions, an apparent carbonyl-region index (CI_app) was calculated as the ratio between the maximum baseline-corrected absorbance in the 1800–1680 cm^−1^ region and the maximum baseline-corrected absorbance in the 3000–2800 cm^−1^ aliphatic C–H stretching region. This index was used only for relative comparison of surface states and was not treated as a direct measure of bulk polymer degradation, because the investigated residues contained interfacial contributions and inorganic particles that may also influence the ATR response.

### 2.3. Thermogravimetric Analysis

Thermogravimetric analysis was performed using a NETZSCH TG 309 Classic thermobalance (NETZSCH-Gerätebau GmbH, Selb, Germany). Samples were placed in open Al_2_O_3_ crucibles with a volume of 85 µL. Measurements were carried out under a nitrogen atmosphere from 30 to 600 °C at a heating rate of 10 K min^−1^ and a gas flow rate of 250 mL min^−1^.

The following parameters were determined: temperature at 5% mass loss (T5%), temperature at 10% mass loss (T10%), temperature at 50% mass loss (T50%), temperature of maximum mass loss rate from the DTG curve (Tmax), maximum DTG value and residual mass at the end of the measurement. For POE-rich residues, the residual mass was interpreted as the combined contribution of inorganic residues, mainly glass and silicon cell fragments, rather than as char formation from POE alone. At the later stage of heating, the sample mass became nearly constant, indicating that no further substantial organic decomposition occurred. Visual inspection of the crucibles after TGA confirmed the presence of thermally stable glass- and silicon-rich residues in selected POE-rich samples.

### 2.4. SEM/EDS Analysis

The surface morphology and local elemental composition of representative POE-rich residues were examined using scanning electron microscopy coupled with energy-dispersive X-ray spectroscopy. The analysis was performed using a ZEISS EVO18 scanning electron microscope (Carl Zeiss AG, Oberkochen, Germany) equipped with a Nano X-flash Detector 5010 EDS detector (Bruker Nano GmbH, Berlin, Germany). Representative L30_V1500 and L30_V2000 samples were selected to assess surface heterogeneity and the presence of inorganic particles attached to or embedded in the polymer-rich fraction. Representative SEM/EDS regions were selected by visual screening of the POE-rich residues to include both polymer-dominated areas and regions containing visible adhered particles. The selection was therefore purposive rather than random and was intended to verify the presence and spatial distribution of inorganic residues suggested by macroscopic inspection and TGA/DTG.

SEM images were acquired at lower magnification to evaluate the general surface morphology and at higher magnification to reveal local particles and surface features. EDS elemental mapping was performed for carbon, oxygen and silicon to distinguish polymer-rich regions from glass- and silicon-rich residues.

Apparent projected area fractions of detectable C, Si and O signals were estimated from the EDS elemental maps using consistent thresholding of the selected fields of view. These values were used only as semi-quantitative descriptors of the spatial distribution of detectable elemental signals and were not interpreted as mass fractions, atomic fractions, absolute elemental concentrations or layer thicknesses.

## 3. Results and Discussion

### 3.1. Macroscopic Heterogeneity and Origin of Inorganic Residues

The analyzed POE-rich residues showed visible heterogeneity, including dark and white regions, locally transparent areas and particles attached to the polymer surface. Representative examples are shown in Figure 1. Macroscopic inspection confirmed the presence of glass and silicon cell fragments in selected samples. Such contamination is expected for real photovoltaic waste fractions, especially when samples are obtained from cut, cracked or mechanically damaged laminate fragments. Interface-derived inorganic debris may affect the consistency and downstream valorization of the recovered POE-rich material and should therefore be minimized through appropriate pre-sorting, controlled handling or post-treatment cleaning.

This observation is important for interpreting both FTIR-ATR and TGA/DTG results. In FTIR-ATR, inorganic residues may contribute to the 1100–1000 cm^−1^ region through Si-O-related bands. In TGA, the same residues increase the residual mass at the end of the measurement. Therefore, the investigated materials should be described as POE-rich residues, not as pure POE.

From a recycling perspective, the presence of inorganic particles may depend strongly on the initial condition of the module. For full-size, intact modules processed before glass fragmentation, the amount of glass-rich contamination in the polymer fraction could potentially be reduced. In contrast, damaged modules, cracked panels or modules affected by hail, storms or mechanical impact are more likely to generate polymer-rich fractions containing glass and silicon cell fragments. This distinction is important when interpreting the practical quality of recovered encapsulant waste streams.

### 3.2. FTIR-ATR Analysis of Reference POE

**Figure 2:**
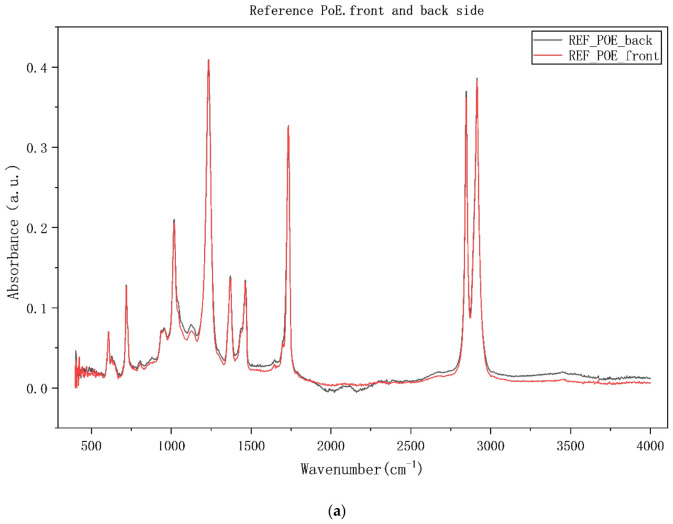

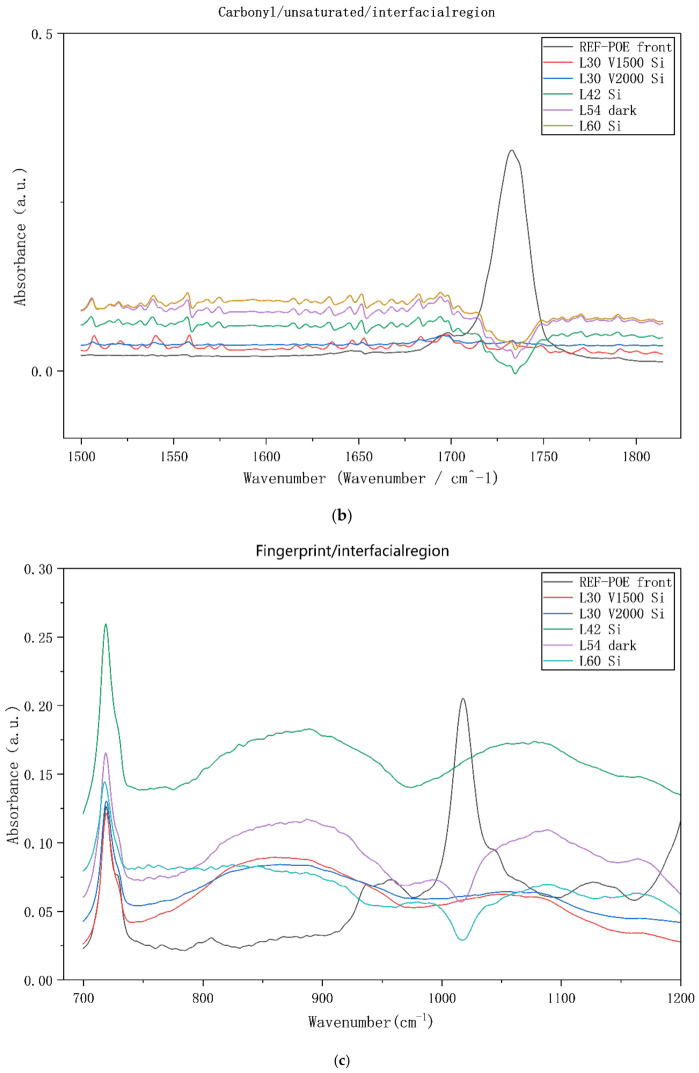
FTIR-ATR spectra of reference POE and representative POE-rich residues: (**a**) full-range spectra of reference POE recorded from the front and back sides; (**b**) enlarged carbonyl/unsaturated/interfacial region between 1800 and 1500 cm^−1^; (**c**) enlarged fingerprint/interfacial region between 1200 and 700 cm^−1^. Spectra in panels (**b**,**c**) were normalized to the aliphatic C–H stretching region to facilitate comparison of local spectral changes.

The reference POE spectra recorded from both sides of the sample showed highly similar spectral profiles, indicating good homogeneity of the baseline material. The close overlap of the front- and back-side spectra is shown in Figure 2a. The characteristic polyolefin bands were observed at approximately 2915 and 2847 cm^−1^, assigned to asymmetric and symmetric stretching vibrations of aliphatic C-H groups, respectively. Additional bands at approximately 1463 cm^−1^ and 1370 cm^−1^ correspond to CH_2_ and CH_3_ deformation vibrations, while the band at approximately 718–719 cm^−1^ is associated with CH_2_ rocking vibrations in polyethylene-like sequences [9,14,24].

A distinct band near 1733 cm^−1^ was also detected in the reference POE. This indicates that carbonyl-containing groups or additives were already present in the baseline material. Therefore, carbonyl-related changes in treated residues should be evaluated through relative intensity, band shape and the appearance of additional components rather than through the mere presence of a C=O band.

### 3.3. FTIR-ATR Analysis of POE-Rich Residues

All POE-rich residues retained the main aliphatic bands characteristic of POE, especially in the 2915–2845 cm^−1^, 1460 cm^−1^ and 718–720 cm^−1^ regions. This confirms that the dominant hydrocarbon backbone of the polyolefin encapsulant remained identifiable after controlled laser treatment. The enlarged spectral regions presented in Figure 2b,c highlight the differences between the reference POE and representative POE-rich residues in the carbonyl/interfacial and fingerprint regions [9,10,11,24,25]. The apparent carbonyl-region index was used to support this comparison and to distinguish relative changes in the carbonyl/interfacial region from the simple presence of a carbonyl band already observed in the reference POE. The FTIR-ATR spectra included additional material states, such as L54_dark and L60_Si, to illustrate the range of surface/interfacial responses observed among the POE-rich residues. The main FTIR-ATR bands considered in the interpretation are summarized in Table 2.

The semi-quantitative CI_app values are summarized in Table 3. The reference POE showed CI_app values of 0.841 and 0.804 for the front and back sides, respectively, reflecting the distinct carbonyl-related band already present in the baseline material. In contrast, the representative POE-rich residues showed considerably lower CI_app values, typically between 0.028 and 0.060. This confirms that the additional features observed in the 1800–1500 cm^−1^ region should not be interpreted as evidence of severe overall thermo-oxidative degradation of the POE matrix. Instead, they indicate localized surface/interfacial modifications superimposed on a largely retained polyolefin spectral profile.

Compared with the reference POE, the POE-rich residues showed greater spectral complexity, particularly in the 1800–1500 cm^−1^ region. In several samples, this region contained additional components near 1770, 1694, 1682, 1651 and 1557 cm^−1^. These bands may originate from carbonyl-containing species, unsaturated structures, surface oxidation products or interfacial residues from glass and silicon cell surfaces. These additional components may result from a superposition of service-induced ageing accumulated during approximately five years of module operation, local thermo-oxidative surface changes and interfacial contributions from glass or silicon cell surfaces. Importantly, these features were observed together with the retained aliphatic POE bands, indicating that the main polyolefin backbone remained identifiable while local chemical and interfacial modifications occurred. Given the heterogeneous nature of the samples, these bands should not be assigned exclusively to degradation of POE.

The 1100–1000 cm^−1^ region also showed notable differences between samples. Bands in this region reflect C-O-containing additives or oxidation products, but they can also be associated with Si-O vibrations from glass, silicate residues or silicon cell-related fragments. This interpretation is consistent with the macroscopic observation of inorganic particles and the residual mass observed in TGA/DTG.

### 3.4. Influence of Surface Region and Interface Type

The FTIR-ATR results showed that the spectral response depended on both the local appearance of the sample and the interface from which the polymer-rich residue originated. Glass-side and silicon-side surfaces exhibited differences in the carbonyl/fingerprint regions, while white and dark regions differed mainly in baseline stability and the contribution of interfacial or inorganic features. The strongest spectral heterogeneity was observed for locally white and dark regions. Some spectra showed non-ideal baselines and bands near 2350 cm^−1^ or in the 2100–2000 cm^−1^ region. These features were not used as primary degradation markers because they may originate from atmospheric/background effects, uneven ATR contact, surface roughness, scattering or local inorganic particles. Therefore, the most reliable interpretation should be based on persistent changes in the carbonyl/fingerprint regions together with the TGA/DTG and SEM/EDS evidence.

### 3.5. TGA/DTG Thermal Stability and Residual Mass

**Figure 3:**
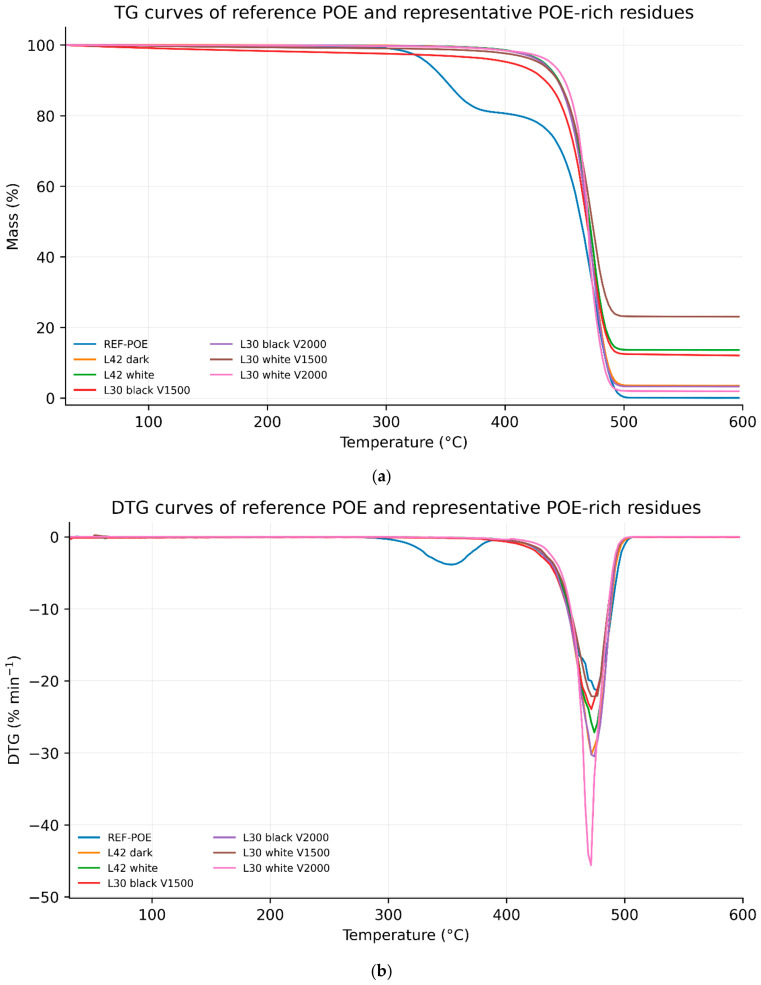

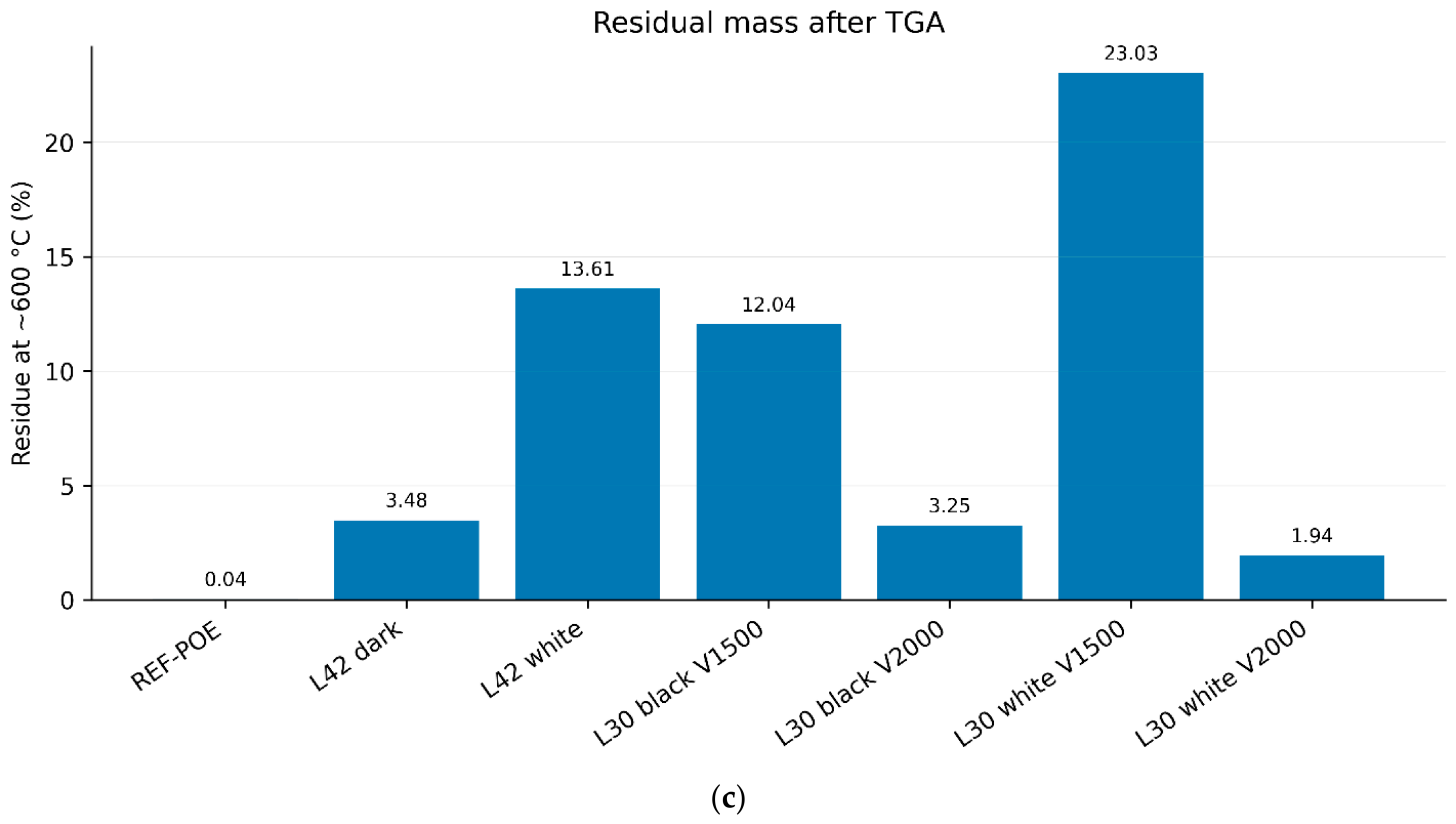
Thermogravimetric behaviour of reference POE and representative POE-rich residues: (**a**) TG curves; (**b**) DTG curves; (**c**) residual mass at approximately 600 °C. The increased residual mass of selected POE-rich residues reflects the presence of inorganic glass- and silicon cell-derived debris rather than char formation from POE alone.

The thermal behaviour of reference POE and representative POE-rich residues is shown in Figure 3, while the extracted TGA/DTG parameters are summarized in Table 4. The reference POE decomposed almost completely under nitrogen, leaving only approximately 0.04% residual mass at the end of the measurement. Its DTG maximum was observed at approximately 476.8 °C.

The earlier apparent mass loss onset of the reference POE compared with several recovered residues should be interpreted in relation to the different material states. The reference sample was an inorganic-free encapsulant film, whereas the recovered POE-rich residues contained thermally stable glass- and silicon cell-derived particles. Since TG curves are normalized to the initial sample mass, the presence of inorganic debris reduces the relative mass fraction of the decomposing polymer and may shift the apparent T5% and T10% values to higher temperatures. In addition, field ageing and previous thermal exposure may have reduced the amount of low-molecular-weight or volatile components contributing to early mass loss.

Similar high-temperature decomposition behaviour has been reported for photovoltaic encapsulant residues and extracted polymer fractions, although direct comparison between EVA and POE should be made cautiously because of their different chemical structures and degradation pathways [26,27,28].

The L42_dark and L42_white samples showed similar main decomposition ranges. For L42_dark, T5%, T10% and T50% were approximately 431.8 °C, 446.7 °C and 471.7 °C, respectively, with a DTG peak at approximately 471.7 °C. For L42_white, the corresponding values were approximately 434.3 °C, 446.7 °C and 471.8 °C, with a DTG peak at approximately 474.3 °C.

These similar decomposition temperatures indicate that the dominant organic POE fraction retained comparable thermal stability in both dark and white regions. The major difference was observed in the residual mass. L42_dark left approximately 3.48% residue, whereas L42_white left approximately 13.61%. Since reference POE left only a trace residue, the increased residual mass in the treated samples is attributed mainly to inorganic contamination, especially glass and silicon cell fragments.

The L30 residues showed the same general one-step decomposition behaviour of the dominant POE fraction, with DTG peak temperatures in the range of approximately 471.5–474.3 °C. However, the onset region and residual mass varied strongly between the dark and white regions and between the two scanning-speed variants. The residual mass ranged from 1.94% for L30_white_V2000_TGA to 23.03% for L30_white_V1500_TGA, confirming that the inorganic fraction was highly localized and depended strongly on the specific sampled region. This variability supports the interpretation that the residual mass is governed primarily by the amount of glass- and silicon cell-derived debris present in the tested fragment rather than by intrinsic char formation from POE.

The higher T5% and T10% values observed for several POE-rich residues compared with reference POE should not be interpreted simply as improved polymer stability. The residues contain inorganic particles that reduce the relative mass fraction of the decomposing organic phase, and previous exposure or sample history may also have removed low-molecular or volatile fractions [26,27,28]. Therefore, the most reliable comparison concerns the main decomposition region, DTG peak position and residual mass. The DTG maxima of the POE-rich residues remained within a similar high-temperature polyolefin decomposition range, while the residual mass clearly reflected the variable contribution of inorganic contamination.

Because the investigated POE-rich residues were heterogeneous fragments of real PV waste material, the TGA/DTG values should be interpreted as descriptive parameters of selected material states rather than as statistically averaged properties of a homogeneous material. Therefore, the present study does not claim statistical significance for small differences in DTG peak temperature. The main comparison is based on the marked differences in residual mass, which were much larger than the small variations in decomposition peak temperature and were supported by macroscopic inspection and SEM/EDS evidence.

### 3.6. SEM/EDS Confirmation of Inorganic Residues

**Figure 4:**
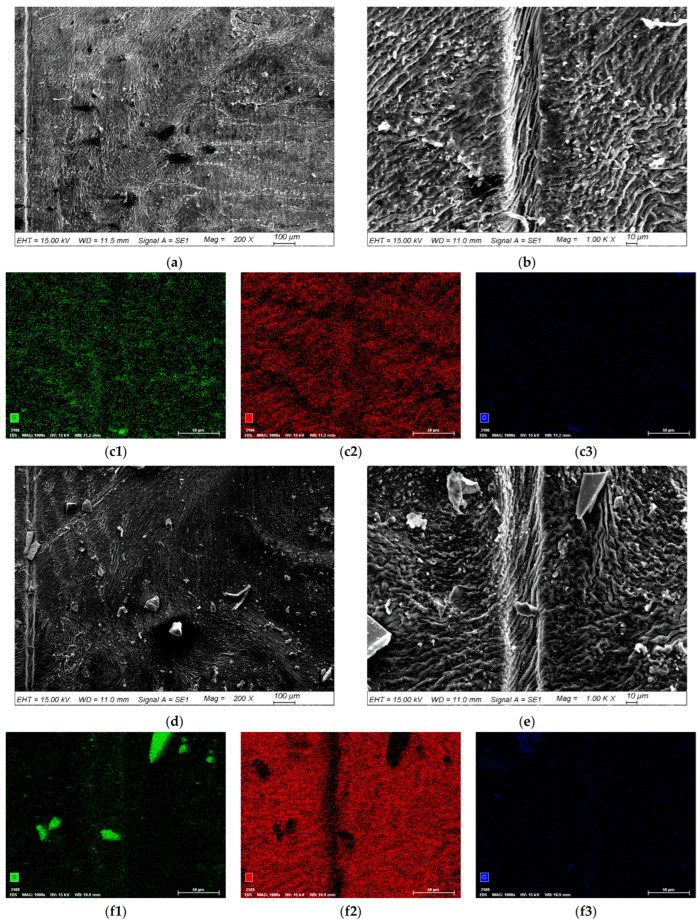
SEM/EDS analysis of representative L30 POE-rich residues. SEM images of L30_V1500 at (**a**) lower and (**b**) higher magnification show a heterogeneous polymer-rich surface with local particles and surface irregularities. Elemental maps of (**c1**) Si, (**c2**) C and (**c3**) O reveal the local distribution of polymer-rich and inorganic regions. SEM images of L30_V2000 at (**d**) lower and (**e**) higher magnification show similar local heterogeneity, while elemental maps of (**f1**) Si, (**f2**) C and (**f3**) O confirm the presence of silicon- and oxygen-containing regions associated with glass or silicon cell residues.

SEM/EDS observations of representative L30_V1500 and L30_V2000 residues are shown in Figure 4. The images revealed heterogeneous polymer-rich surfaces with local roughness, grooves, adhered particles and embedded fragments. The observed morphology confirms that the analyzed material should be treated as a real POE-rich waste fraction rather than as a chemically purified polymer film.

EDS elemental mapping showed that carbon-rich regions were associated with the polymer-dominated matrix, whereas local silicon- and oxygen-containing areas corresponded to inorganic particles attached to or embedded in the residue. Semi-quantitative image analysis of the EDS maps further confirmed the heterogeneous distribution of detectable elemental signals. In the area shown in Figure 4(c1–c3), the apparent projected area fraction of the carbon signal was approximately 90.3%, whereas the corresponding values for silicon and oxygen were approximately 5.9% and 4.8%, respectively. In contrast, the area shown in Figure 4(f1–f3) exhibited a lower apparent carbon signal fraction of approximately 65.1% and a higher silicon signal fraction of approximately 32.2%, with oxygen at approximately 3.0%. These values indicate that the selected fields of view differed strongly in the local contribution of carbon-rich polymer-dominated regions and Si-containing inorganic particles. Because the maps were evaluated as projected signal areas, these values should not be interpreted as mass fractions, atomic fractions, absolute elemental concentrations or layer thicknesses. The values were obtained from independently thresholded elemental maps and should not be expected to sum exactly to 100%.

These regions are consistent with glass- or silicon cell-derived fragments observed during sample preparation. Although SEM/EDS was performed on representative L30 residues rather than directly on the L42 samples used for TGA, the L30 and L42 residues showed comparable macroscopic heterogeneity, including polymer-rich areas with locally adhered inorganic particles. Direct one-to-one correlation between SEM/EDS and TGA specimens was not possible because TGA is destructive and requires the consumption of small sample fragments. Therefore, SEM/EDS results should be interpreted as supporting evidence for the presence of inorganic debris in the POE-rich waste stream rather than as a direct elemental quantification of each TGA specimen.

Together with macroscopic inspection and TGA/DTG results, SEM/EDS supports the conclusion that the quality of recovered POE-rich residues depends not only on the preservation of the polyolefin matrix, but also on the amount and distribution of glass- and silicon-derived particles.

### 3.7. Implications for Recycling and Valorization

The combined FTIR-ATR, TGA/DTG and SEM/EDS results indicate that POE-rich encapsulant residues can preserve the main polyolefin character while containing pronounced and locally variable inorganic contamination. This has direct implications for downstream valorization, because the quality of the recovered polymer-rich fraction depends not only on the chemical state of POE, but also on the amount and distribution of glass and silicon cell particles [13,15,16,27,28]. During potential melt-reprocessing, rigid inorganic particles may act as stress concentrators and may affect the mechanical and rheological stability of the recyclate, as commonly observed in polymer systems containing hard particulate contaminants or fillers. However, mechanical and rheological testing was outside the scope of the present work. Therefore, this aspect should be addressed in future valorization studies.

For intact full-size modules, contamination by glass fragments could potentially be minimized if layer separation occurs before extensive mechanical fragmentation. In contrast, waste streams containing cracked, storm-damaged or mechanically broken modules are more likely to generate polymer-rich fractions contaminated with glass and silicon cell fragments. Therefore, downstream recycling strategies should consider pre-classification of module condition and possible cleaning or separation steps before polymer valorization.

## 4. Conclusions

1. Reference POE showed characteristic polyolefin FTIR-ATR bands at approximately 2915–2847 cm^−1^, 1463 cm^−1^ and 718–719 cm^−1^. A carbonyl-related band near 1733 cm^−1^ was already present in the reference material, indicating that degradation assessment should not rely solely on the presence of this band.
2. POE-rich residues retained the main spectral features of the polyolefin backbone, confirming that the dominant POE structure remained identifiable after controlled laser treatment of photovoltaic laminate fragments.
3. Local spectral differences were observed mainly in the 1800–1500 cm^−1^ and 1100–1000 cm^−1^ regions. These differences reflect a combination of surface modification, oxygen-containing species and interfacial inorganic residues.
4. TGA/DTG analysis showed that the dominant POE-rich fraction in the investigated residues decomposed within a similar high-temperature range, with DTG peak temperatures mainly between approximately 471.5 and 474.3 °C. This suggests comparable thermal behaviour of the dominant organic POE fraction in the analyzed residue states.
5. The main differences between the analyzed residues were observed in residual mass after TGA. The residue at approximately 600 °C ranged from 1.94% to 23.03% for POE-rich residues, compared with only 0.04% for reference POE. This confirms that inorganic residues substantially influence the thermal analysis of real POE-rich waste fractions.
6. The presence of glass and silicon cell fragments should be considered a key factor in assessing the quality of recovered POE-rich residues. Such contamination may be minimized for intact full-size modules processed before extensive glass fragmentation, but it is likely to be more significant for damaged, cracked or mechanically broken photovoltaic panels.
7. Future valorization strategies for POE-rich encapsulant waste should combine assessment of polymer chemical stability with quantitative evaluation of inorganic contamination, because the mechanical and rheological quality of the recyclate may be affected by the presence and distribution of hard glass- and silicon-derived particles.

## Figures and Tables

**Table 1:** Sample nomenclature, material region and analytical use of the investigated POE-rich residues.

Sample Code	Material/Region Represented	Nominal Cumulative Fluence [J cm^−2^]	Analysis Performed
REF_POE_front/REF_POE_back	Reference POE film, front and back sides	—	FTIR-ATR
L30_V1500_Si	POE-rich residue, silicon cell-facing side	75.0	FTIR-ATR
L30_V2000_Si	POE-rich residue, silicon cell-facing side	56.3	FTIR-ATR
L42_Si	POE-rich residue, silicon cell-facing side	157.5	FTIR-ATR
L54_dark	POE-rich residue, visually dark region	101.3	FTIR-ATR
L60_Si	POE-rich residue, silicon cell-facing side	250.0	FTIR-ATR
L42_dark	POE-rich residue, visually dark region	157.5	TGA/DTG
L42_white	POE-rich residue, visually white region	157.5	TGA/DTG
L30_black_V1500	POE-rich residue, visually dark/black region	75.0	TGA/DTG
L30_white_V1500	POE-rich residue, visually white region	75.0	TGA/DTG
L30_black_V2000	POE-rich residue, visually dark/black region	56.3	TGA/DTG
L30_white_V2000	POE-rich residue, visually white region	56.3	TGA/DTG
L30_V1500	Representative POE-rich residue	75.0	SEM/EDS
L30_V2000	Representative POE-rich residue	56.3	SEM/EDS

The L-number in the sample code denotes the nominal average-power code, whereas V1500 and V2000 denote scan-speed variants of 1500 and 2000 mm s^−1^, respectively. Samples without a V suffix were prepared using the common scan-speed condition applied in the corresponding experimental series. The nominal cumulative fluence was calculated from the average laser power, scanning speed, number of passes and line spacing and was used only as a compact descriptor of the exposure level. It should not be interpreted as a complete laser-processing recipe or process-optimization parameter set.

**Table 2:** Main FTIR-ATR bands used for assignment of reference POE and POE-rich residues.

Wavenumber/Region [cm^−1^]	Possible Assignment	Interpretation in This Work	Suggested References
2915–2847	asymmetric and symmetric C–H stretching of CH_2_ groups	retained aliphatic POE backbone	[9,14,24]
~1463 and ~1370	CH_2_ bending and CH_3_ deformation vibrations	characteristic polyolefin features	[9,14]
~1733	carbonyl-related band	already present in reference POE; not sufficient alone as degradation marker	[9,10,24,25]
1770–1680	carbonyl-containing species, oxidized groups, unsaturated/interfacial contributions	local ageing, thermo-oxidative surface changes and interfacial residues	[9,10,11,24,25]
~1651 and ~1557	unsaturated structures, carboxylate-like species or interfacial residues	cautiously interpreted; not assigned exclusively to POE degradation	[10,14,24,25]
1100–1000	C–O contributions and/or Si–O vibrations	additives/oxidation products and glass/silicate/silicon cell-related residues	[14,27,28]
718–720	CH_2_ rocking in polyethylene-like sequences	retained polyethylene-like segments in POE	[9,14]

**Table 3:** Apparent carbonyl-region index calculated from FTIR-ATR spectra of reference POE and representative POE-rich residues.

Sample	Carbonyl/Interfacial Peak Position [cm^−1^]	C–H Reference Peak Position [cm^−1^]	CI_app
REF_POE_front	1733.2	2914.9	0.841
REF_POE_back	1733.2	2914.4	0.804
L30_V1500_Si	1698.5	2915.8	0.037
L30_V2000_Si	1695.1	2916.3	0.028
L42_Si	1694.6	2913.9	0.038
L54_dark_rep1	1694.6	2913.4	0.052
L60_Si_rep1	1694.2	2913.4	0.060
L60_Si_rep2	1694.6	2913.9	0.054

CI_app was calculated using baseline-corrected peak heights. The index was used for relative comparison only and should not be interpreted as a direct quantitative measure of bulk POE degradation. Spectra with unstable C–H normalization after baseline correction were excluded from the semi-quantitative comparison.

**Table 4:** TGA/DTG parameters of reference POE and representative POE-rich residues measured under nitrogen atmosphere.

Sample	Sample Mass [mg]	T5% [°C]	T10% [°C]	T50% [°C]	Tmax DTG [°C]	DTG Peak [% min^−1^]	Residue at ~600 °C [%]
REF_POE	12.182	336.83	351.83	464.20	476.80	−21.29	0.04
L42_dark	11.132	431.78	446.73	471.73	471.73	−30.25	3.48
L42_white	9.974	434.25	446.74	471.75	474.27	−27.15	13.61
L30_black_V1500	10.713	404.31	434.28	469.23	471.76	−23.92	12.04
L30_white_V1500	11.740	429.28	446.76	474.27	474.27	−22.18	23.03
L30_black_V2000	11.096	431.78	446.74	471.74	474.27	−30.50	3.25
L30_white_V2000	11.966	439.25	451.72	471.49	471.49	−45.61	1.94

## Data Availability

The original contributions presented in this study are included in the article. Further inquiries can be directed to the corresponding author(s).

## Abbreviations

The following abbreviations are used in this manuscript:

ATR	attenuated total reflectance
DTG	derivative thermogravimetry
EDS	energy-dispersive X-ray spectroscopy
EoL	end-of-life
FTIR	Fourier-transform infrared spectroscopy
POE	polyolefin elastomer
PV	photovoltaic
SEM	scanning electron microscopy
TGA	thermogravimetric analysis
